# Azithromycin resistance in nontyphoidal *Salmonella* in an urban informal settlement in Nairobi, Kenya

**DOI:** 10.1128/spectrum.00985-25

**Published:** 2025-10-27

**Authors:** Kelvin Kering, Kariuki Njaanake, Celestine Wairimu, Marianne Mureithi, Collins Kebenei, Cecilia Mbae, Michael Mugo, Susan Kavai, Evans Kibet, Winfred Mbithi, Kristin Weber, Michael Pietsch, Oliver Drechsel, Torsten Semmler, Andrea Thurmer, Antje Flieger, Lothar H. Wieler, Samuel Kariuki

**Affiliations:** 1 Centre for Microbiology Research, Kenya Medical Research Institute 118982, Nairobi, Kenya; 2 Department of Medical Microbiology and Immunology, University of Nairobi 107854, Nairobi, Kenya; 3 Robert Koch Institute 235859 , Wernigerode, Germany; 4 Robert Koch Institute 9222, Berlin, Germany; 5 Digital Global Public Health, Hasso Plattner Institute, Universitat Potsdam 26583, Potsdam, Germany; 6 Drugs for Neglected Diseases initiative, Nairobi, Kenya; University of Saskatchewan, Saskatoon, Saskatchewan, Canada

**Keywords:** Kenya, azithromycin, nontyphoidal *Salmonella*, antimicrobial resistance

## Abstract

Nontyphoidal *Salmonella* (NTS) poses a significant burden of bacteremia in sub-Saharan Africa (SSA). Antimicrobial resistance (AMR) complicates the management of NTS infections. The NTS carriage of mobile genetic elements harboring AMR genes can result in the spread of resistance. We investigated the AMR profiles and genomic characteristics of 74 NTS isolates recovered from children under 5 years of age during acute disease, post-treatment shedding, and in healthy carriers. Whole genome sequencing data were used to identify AMR genes, plasmid replicons, and virulence determinants. Of the 74 NTS isolates, 63.5% (47) were *Salmonella* Enteritidis, the rest being *Salmonella* Typhimurium. More than a third, 37.8% (2874), were resistant to at least one antibiotic. A small proportion, 8.1% (6/74), were multidrug resistant, with a majority (5/6) being *S*. Typhimurium. Resistance to azithromycin, one of the current drugs of choice for treating invasive nontyphoidal *Salmonella* (iNTS), was observed in 13.5% (10/74) of the isolates. Notably, the macrolide resistance gene *mph(A*) was detected in one isolate. Extended-spectrum β-lactamase (ESBL) genes (*bla*_CTX-M-3_ and *bla*_TEM_) were identified in 8.1% of the isolates, all of which were *S*. Typhimurium. Six plasmid replicons were detected, with epidemic plasmids IncFIB(S)_1 and IncFII(S)_1 observed in all isolates. The ESBL gene *bla*_CTX-M-3_ was harbored in the IncI1_1_Alpha plasmid replicon. A total of 117 virulence determinants were identified, with only one isolate carrying the *shdA* gene associated with prolonged fecal shedding. Resistance and reduced susceptibility of NTS to antibiotics could result in treatment failure and underscore the need for continued surveillance, vaccine introduction, and antimicrobial stewardship.

**IMPORTANCE:**

Antimicrobial resistance is a huge public health concern globally, particularly in low-resource settings due to a high burden of infectious diseases, limited access to quality healthcare, and misuse and/or overuse of antibiotics. Azithromycin is one of the antibiotics used in the treatment of iNTS; notably, 13.5% of the NTS isolates in this study showed resistance to azithromycin. The detection of AMR determinants and plasmids highlights the possibility of these determinants spreading, resulting in increased resistance. Resistance of NTS to azithromycin and other antibiotics in this setting could lead to treatment failure, resulting in poor patient outcomes. These findings emphasize the need for vaccine introduction and antimicrobial stewardship in health facilities in low-resource settings.

## INTRODUCTION

Nontyphoidal *Salmonella* serovars are common foodborne pathogens globally responsible for mostly uncomplicated mild diarrhea in immunocompetent individuals (1–3). Among the immunocompromised, the malnourished, the elderly individuals, and children under 5 years, NTS can become invasive, leading to life-threatening disease (4). When not managed promptly or left untreated, iNTS disease can result in high mortality, with previous studies reporting annual case fatality rates of approximately 15% among all ages globally (5, 6) and 17.1% in Africa (2). A huge proportion (78.9% or 422,000 cases) of the global iNTS disease and mortalities (~86%, 66,500 deaths) were previously reported in SSA (5), with mortality rates observed among children under 5, elderly persons (≥70 years), and HIV-infected persons (5). In Kenya, the incidence of iNTS among children below 5 years has previously been reported to be 997.9 per 100,000 person-years of observation in an urban setting (7).

Two NTS serovars, *Salmonella* Typhimurium and *Salmonella* Enteritidis, are responsible for the majority of the iNTS disease reported in SSA. In particular, *S*. Typhimurium sequence type (ST)313 and *S*. Enteritidis ST11 are predominantly implicated in NTS infections in SSA (8, 9). *S*. Typhimurium ST19 is also a common cause of NTS infections in SSA (10). Currently, three antibiotics (ciprofloxacin, ceftriaxone, and azithromycin) are recommended for the management of NTS infections (11, 12). The emergence of multidrug-resistant (MDR) NTS strains with resistance to extended-spectrum β-lactams and fluoroquinolones has complicated treatment, resulting in longer hospitalization, the need for more expensive last-line antibiotics, and poor outcomes for patients, especially in SSA and Asia (13–16). Several studies have reported on MDR NTS in SSA (17), including in Kenya (3, 4), Mozambique (18), Malawi (19, 20), Nigeria (21), and the Democratic Republic of Congo (22), with a high proportion of the MDR NTS being *S*. Typhimurium.

The emergence and spread of MDR NTS strains have been associated with the horizontal spread of antibiotic resistance genes through plasmids. Different studies in SSA (13, 21–24) have documented the presence of several resistance genes conferring resistance to different antibiotics, including fluoroquinolones, macrolides, and β-lactams. In addition, several reports have shown that iNTS strains exhibiting resistance to extended-spectrum β-lactams, including ceftriaxone, harbor IncHI2 plasmids (13, 25), which contain the *bla*_CTX-M_ and *bla*_TEM_ genes (26). Of concern are plasmids harboring virulence and resistance determinants such as the IncI1 virulence-resistance plasmid (10, 25, 27), which confer the capability of systemic dissemination. Whereas NTS serovars possess different virulence factors, the presence or absence of these factors has an implication on the virulence of the strains and manifestation of disease (27).

NTS is a zoonotic, foodborne, self-limiting infection in developed countries, but in SSA, NTS is thought to be transmitted mainly through human-to-human contact, and carriers play a critical role (28). It is therefore crucial to understand the virulence and AMR determinants of NTS since this could influence disease outcomes in high-risk populations. Given the increasing rates of MDR NTS and emerging resistance to drugs of last resort, there is a need to understand the genetic determinants influencing the high resistance, especially in areas such as informal settlements, where the likelihood for dissemination of NTS strains and antibiotic resistance determinants is high. This information would help in developing appropriate intervention measures and guiding patient management, especially in facilities with limited laboratory capabilities. This study presents follow-on findings of previous work (29) on resistance and reduced susceptibility of NTS to antibiotics of choice for treatment of iNTS, plasmid types, and virulence genes in *S*. Typhimurium and *S*. Enteritidis isolated from index cases (patients) and asymptomatic carriers from Mukuru urban informal settlement in Nairobi, Kenya.

## MATERIALS AND METHODS

This study utilized clinical NTS (*S*. Typhimurium and *S*. Enteritidis) isolates obtained from a previous study (29) conducted from June 2021 to August 2023. The description of the parent study setting and population, participant enrollment, sample collection, and laboratory analysis is detailed in a previous report (29).

### Description of study site and population

The study was conducted in an urban slum (Mukuru Informal Settlement) located ca. 15 km east of Nairobi city center. The populous informal settlement has limited access to water and dilapidated sanitation and hygiene infrastructure, which expose the residents to infectious diseases, especially enteric diseases. The study population was children under 5 years presenting with fever or a history of fever (axillary temperature of ≥38°C) for more than 24 hours with or without diarrhea (three or more episodes of loose or watery stool in the preceding 24 hours) and healthy individuals in the community.

### Recruitment of study participants and sample collection

Eligible participants presenting at the recruiting health facilities were enrolled, and blood (1–3 mL) and stool/rectal swabs were collected as described previously (29). The samples were transported to the Kenya Medical Research Institute Microbiology Laboratory within ca. 4–5 hours of collection. In addition, stool samples were also collected from the index cases (children) post-treatment to check for NTS shedding. Healthy individuals of all ages living with index cases (contacts) and living in a household 100 m from the index case (controls) were also enrolled and requested to provide stool for isolation of NTS.

### Laboratory analysis

As described elsewhere (4), blood culture bottles were incubated at 37°C for 24 hours in a BACTEC 9050 Blood Culture System (BD, Franklin Lakes, NJ, USA) and subcultured onto MacConkey, blood, and chocolate agar (Oxoid, Basingstoke, UK). Fecal samples were enriched in selenite fecal broth (Oxoid) at 37°C overnight and then subcultured on MacConkey and xylose lysine deoxycholate (Oxoid) agar at the same conditions. Presumed *Salmonella* colonies were subcultured on Mueller-Hinton agar (Oxoid) and identified through biochemical characterization using API 20E strips (Biomerieux, Marcy-l'Étoile, France) and serotyped with specific antisera (Remel; Thermo Fisher Scientific, Massachusetts, USA).

### Antimicrobial susceptibility testing

Seventy-four NTS isolates were subjected to antibiotic susceptibility testing using the Kirby-Bauer disk diffusion technique. Of the 74 isolates, 3 (all *S*. Enteritidis) were recovered from blood and 71 from stool of both cases and among asymptomatic individuals shedding NTS. The antimicrobial susceptibility testing was done three independent times, and the results were averaged. The cut-off for the difference in readings between the three experiments was ±1–2 mm, with outlier readings repeated or excluded. Briefly, the NTS isolates were subcultured onto tryptone soy agar (Oxoid) to obtain pure colonies. A loop full of the colony was emulsified in normal saline to make a suspension with the 0.5 MacFarland standard used as reference for the suspension. A sterile cotton-tipped swab was used to pick the suspension and plate it onto Mueller-Hinton agar (Oxoid). A disk dispenser was used to dispense the antibiotics onto the media in petri dishes of 90 mm in diameter. The antibiotics used included amoxicillin-clavulanic acid (30 µg), ampicillin (10 µg), azithromycin (15 µg), cefotaxime (30 µg), cefpodoxime (10 µg), ceftazidime (30 µg), ceftriaxone (30 µg), chloramphenicol (30 µg), ciprofloxacin (5 µg), nalidixic acid (30 µg), kanamycin (30 µg), gentamicin (10 µg), sulfamethoxazole-trimethoprim (25 µg), tetracycline (30 µg), and streptomycin (10 µg), all from Oxoid. As control organisms, American Type Culture Collection *Escherichia coli* 25922 and *Staphylococcus aureus* 25923 were also subjected to antibiotic susceptibility testing. Result interpretation was based on Clinical and Laboratory Standards Institute guidelines (M100) (34th Ed.) (30). For azithromycin, the cut-off points used were ≥13 as susceptible and ≤12 as resistant. For streptomycin, the cut-off points used were ≥15 mm as susceptible, 12–14 mm as intermediate, and ≤11 mm as resistant. At minimum, multidrug resistance was defined as resistance to at least one antibiotic from three different antibiotic classes.

### Whole genome sequencing and analysis

Genomic DNA extracted using Quick-DNA Fungal/Bacterial Miniprep Kit (Zymo Research) was subjected to whole genome sequencing (WGS) using the Illumina NextSeq 2000 platform at the Genome Competence Center, Robert Koch Institute in Berlin. Low-quality bases were trimmed from the raw reads using fastp (31), and *de novo* assembly was done through SPAdes (version 1.1.0) (32). Antimicrobial resistance and virulence genes were identified through the Abricate software (version 1.0.1) (33) using the Resfinder database (version 2023-11-04) and Virulence Factor Database (version 2023-11-04), respectively, while plasmids were identified using the PlasmidFinder database (version 2023-11-04). In addition, the Comprehensive Antibiotic Resistance Database (CARD) was also used to identify AMR genes. Genes and plasmid replicons with 80% coverage and 80% identity were considered present. MOB-Suite (34) was used to identify the AMR genes carried by plasmids (https://www.solugenomics.com/tools/mob-suite).

## RESULTS

Of the 74 NTS (*S*. Typhimurium and *S*. Enteritidis) isolates analyzed in this study, 44 were isolated from index cases during acute disease, while 25 isolates were recovered during post-treatment shedding and 5 isolates from asymptomatic carriers (healthy individuals). The majority (63.5%, 47/74) of the isolates were *S*. Enteritidis. All the *S*. Enteritidis were of sequence type 11, while all *S*. Typhimurium were of sequence type 19.

### Antibiotic susceptibility profiles

**Fig 1:**
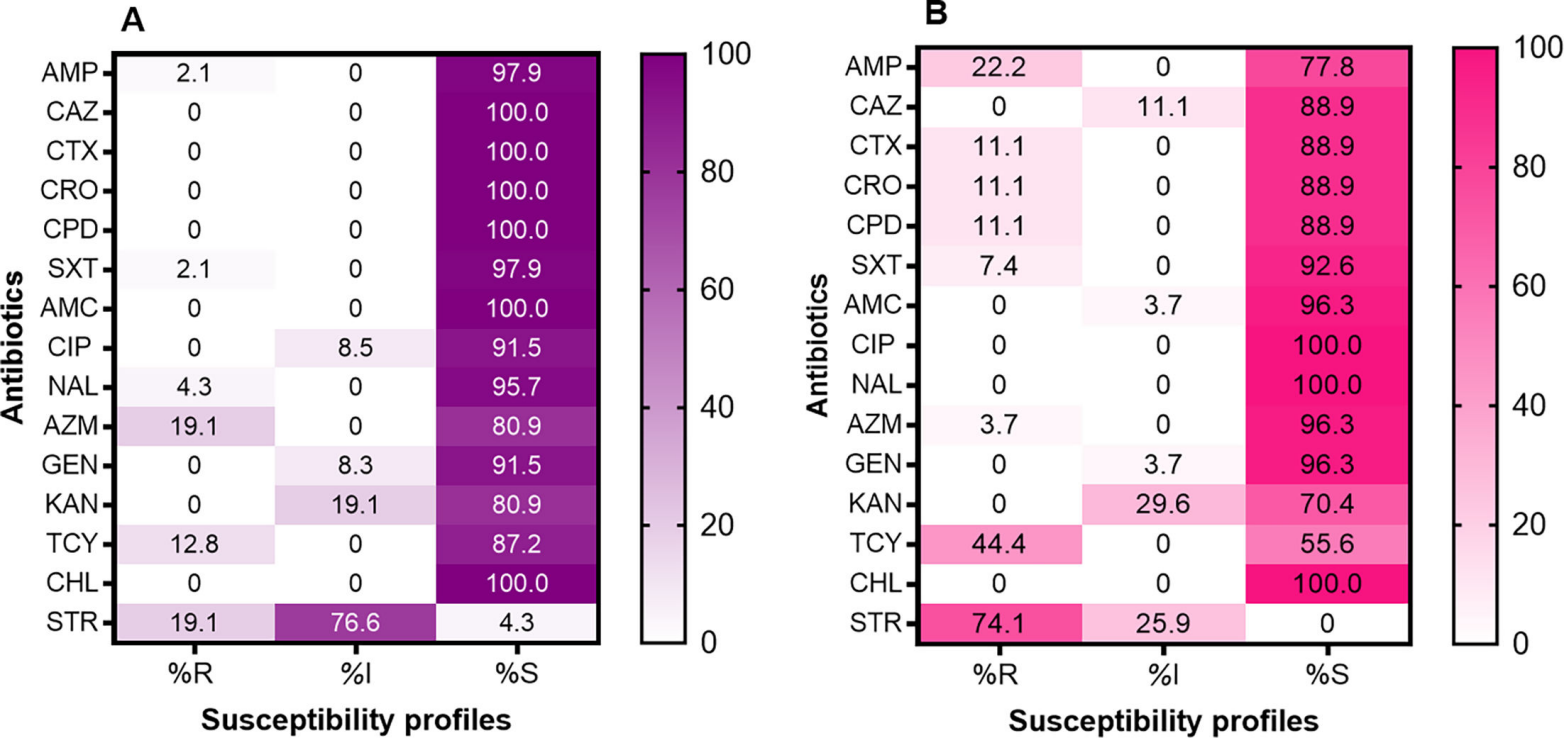
Percentage of antibiotic susceptibility profiles of (**A**) 47 S. Enteritidis and (**B**) 27 S. Typhimurium isolates. The color coding indicates the level of susceptibility to antibiotics. AMC, amoxicillin-clavulanic acid; AMP, ampicillin; AZM, azithromycin; CAZ, ceftazidime; CHL, chloramphenicol; CIP, ciprofloxacin; CPD, cefpodoxime; CRO, ceftriaxone; CTX, cefotaxime; GEN, gentamicin; I, intermediate; KAN, kanamycin; NAL, nalidixic acid; R, resistant; S, sensitive; STR, streptomycin; SXT, sulfamethoxazole-trimethoprim; TCY, tetracycline.

Antibiotic resistance, and in particular multidrug resistance, of NTS is likely to complicate patient management in this setting, which could lead to treatment failure. In this study, a small proportion (8.1%) of the isolates were MDR (resistant to one or more antibiotics in three different classes), while 37.8% (28/74) were resistant to at least one antibiotic. The majority (five out of six) of the MDR isolates were *S*. Typhimurium. Resistance to ampicillin, sulfamethoxazole-trimethoprim, and tetracycline was 9.5% (7/74), 4.05% (3/74), and 24.3% (18/74), respectively. Of the 74 isolates, resistance to azithromycin was observed in 13.5% (10). Azithromycin resistance was higher in *S*. Enteritidis (19.1%) compared to *S*. Typhimurium (3.7%) (Fig. 1). Three (11.1%) of the 27 *S*. Typhimurium isolates were each resistant to ceftriaxone, cefotaxime, and cefpodoxime (Fig. 1B). Noteworthy is that the three isolates were recovered from the same patient during acute disease and post-treatment shedding. All *S*. Typhimurium isolates in this study were susceptible to ciprofloxacin; however, 8.5% (447) of *S*. Enteritidis showed reduced susceptibility.

All the isolates in this study were susceptible to chloramphenicol (Fig. 1). The resistance phenotype ampicillin/cefotaxime/ceftriaxone/cefpodoxime/tetracycline was observed in three *S*. Typhimurium isolates recovered from the same child during acute disease and post-treatment shedding. One *S*. Enteritidis isolate recovered from an index case during acute disease had the resistance phenotype ampicillin/sulfamethoxazole-trimethoprim/azithromycin and tetracycline.

One observation from the AMR patterns of isolates recovered during acute disease, post-treatment shedding, and from asymptomatic carriers is that four isolates recovered during acute disease were MDR, compared to two during post-treatment shedding and none from carriers. A comparison of resistance to azithromycin during acute disease and post-treatment shedding was observed to be 15.9% (7/44) and 12% (3/25), respectively. Resistance profiles of isolates recovered during acute disease did not significantly differ from those of post-treatment shedding (*P* > 0.05). The majority of five isolates recovered from asymptomatic carriers were susceptible to most antibiotics, with two of the isolates being resistant to tetracycline and one showing reduced susceptibility to ciprofloxacin.

### Antibiotic resistance genes

A diverse spectrum of AMR genes was detected in this study, highlighting the possible widespread distribution of genes conferring resistance to different antibiotics. Based on WGS data, a total of 14 AMR genes were identified in different NTS isolates from the ResFinder database. The most common AMR genes were those conferring resistance to aminoglycosides, sulfonamides, and tetracycline. All the 74 isolates harbored the chromosomally encoded aminoglycoside resistance gene *aac(6′)-Iaa*. In addition, 21.6% (16/74) of the isolates carried the aminoglycoside genes *aph(3″)-Ib* and *aph(6)-Id,* while one isolate possessed the gene *ant(3″)-Ia* (Table 1). The sulfonamide resistance genes, *sul1* and *sul2*, were detected in 1 and 14 isolates, respectively, while trimethoprim (*dfrA1* and *dfrA8*) resistance genes were each present in one isolate. The tetracycline resistance gene *tetA* was present in 21.6% (16/74) of the isolates, while the macrolide resistance gene *mph*(A) was detected in one isolate. However, using the CARD database, the genes *emrA*, *emrB*, and *tolc*, which are associated with efflux pump-mediated resistance, were detected in all the isolates. Extended-spectrum β-lactamase (ESBL) genes were identified in 8.1% (674) of the isolates, and all six isolates harboring ESBL genes were *S*. Typhimurium. ESBL gene *bla*_CTX-M-3_ was detected in three *S*. Typhimurium isolates recovered from the same patient during acute disease and post-treatment shedding. The genes *bla*_TEM-135_, *bla*_TEM-1B_, and *bla*_TEM-1C_ were present in one isolate each. One isolate (*S*. Typhimurium) had nine resistance genes, including *bla*_TEM-1C_, while another *S*. Typhimurium isolate carried eight genes, including *bla*_TEM-1B._

**TABLE 1:** Distribution of antibiotic resistance genes between *S. Typhimurium* and *S. Enteritidis* identified from the ResFinder database

Antibiotic resistance gene	Frequency of occurrence	
	*S. Typhimurium* (*N* = 27) *n* (%)	*S. Enteritidis* (*N* = 47) *n* (%)
*aac(6′)*-*Iaa*	27 (100)	47 (100)
*ant(3″)*-*Ia*	1 (3.7)	0
*aph(3″)*-*Ib*	16 (59.3)	0
*aph(6)-Id*	16 (59.3)	0
*bla*_CTX-M-3_	3 (11.1)	0
*bla*_TEM-135_	1 (3.7)	0
*bla*_TEM-1B_	1 (3.7)	0
*bla*_TEM-1C_	1 (3.7)	0
*dfrA1*	1 (3.7)	0
*dfrA8*	1 (3.7)	0
*sul1*	1 (3.7)	0
*sul2*	14 (51.9)	0
*tetA*	16 (59.3)	0
*mphA*	1 (3.7)	0

### Plasmid replicon types

Plasmids are likely to contribute to the transfer of AMR genes between isolates, thus contributing to an increase in AMR. Six types of plasmid replicons (Col8282_1, ColRNAI_1, IncFIB(S)_1, IncFII(S)_1, IncI1_1_Alpha, and IncQ1_1) were identified in different isolates based on WGS analysis on the PlasmidFinder tool. IncFIB(S)_1 and IncFII(S)_1 were the predominant plasmid types detected and were present in all 74 isolates. The conjugative plasmid IncI1_1_Alpha was present in five isolates, all of which were *S*. Typhimurium, and four were multidrug resistant. Of the four multidrug-resistant isolates, in three isolates the plasmid IncI1_1_Alpha carried the AMR gene *bla*_CTX-M-3_, while in one isolate the plasmid carried five AMR genes including *sul1*, *dfrA1*, *tetA*, *ant(3″)-Ia*, and *bla*_TEM-1C_. Col8282_1 was present in one (*S*. Typhimurium) isolate, while ColRNAI_1 was detected in 20 (27%) of the 74 isolates, all of which were *S*. Typhimurium. The plasmid replicon IncQ1_1 was detected in only one *S*. Typhimurium isolate.

### Distribution of virulence determinants

The isolates in this study contained a huge diversity of virulence determinants. Based on WGS analysis in the Virulence Factor Database, 117 virulence determinants were identified in all the isolates. Predictably, genes located on *Salmonella* pathogen island (SPI) SPI-1, SPI-2, SPI-3, and SPI-5 were present in the majority of the isolates. The majority (88.03%, 103/117) of the virulence genes detected were present in all the isolates. Some of the virulence genes present in all the 74 isolates include csg fimbrial gene cluster *csgA* to *csgG*, fim gene cluster (*fimC*, *fimD*, *fimF*, *fimH*, and *fimI*), and invasion inv gene cluster (*invA* to *invJ*). The genes *avrA*, *rcK*, *spvB*, *spvC*, and *spvR* were identified in all isolates except one *S*. Typhimurium isolate. In addition, the SPI-1- and SPI-2-encoded *sseK1* and *sseK2* effector genes were detected in 59.5% (44/74) and 33.8% (25/74) of the isolates, respectively. The Gifsy-1 prophage-encoded gene *gogB* was present in nine (12.2%) isolates, all of which were *S*. Typhimurium. Notably, the *shdA* gene was present in one *S*. Typhimurium isolate, which was lacking several genes, including *gogB*, *avrA*, *rcK*, *spvB*, *spvC*, *spvR*, and *grvA.* The detection of a huge proportion of conserved genes in both serotypes indicates the importance of these virulence genes in NTS pathogenesis.

## DISCUSSION

Nontyphoidal *Salmonella* (invasive and gastroenteritis) disease continues to be a significant health concern in SSA settings, especially among children and in areas where it is endemic. This is worsened by asymptomatic shedding, which could result in the spread of infections and an increase in antibiotic-resistant NTS strains in the community. The majority (63.5%, 47/74) of the isolates recovered from children during acute disease (fever with/without diarrhea) and post-treatment shedding and among healthy carriers were *S*. Enteritidis. This finding differs from previous reports where *S*. Typhimurium has often been the predominant serovar recovered in Kenya (4, 13) and in other countries (23, 35–38). Consequently, these findings highlight the evolving epidemiology of NTS disease in this setting, which could influence vaccine development.

A wide spectrum of resistance was observed with the highest prevalence in *S*. Typhimurium resistance to tetracycline (44.4%, 12/27). In contrast to a previous study (4) in the same study setting, which observed that 68.3% of iNTS isolates were resistant to at least one antibiotic in 2019, we found that 37.8% of the isolates were resistant to at least one antibiotic. This could be attributed to the fact that *S*. Typhimurium ST313, which was previously the dominant ST in this setting, was not detected in this study. Of concern is the resistance (13.5%) of NTS to azithromycin, one of the current antibiotics of choice for management of iNTS. Increase in azithromycin resistance could lead to treatment failure within this setting, as routine laboratory testing in healthcare facilities is not available; hence, patients are managed empirically. Interestingly, azithromycin resistance in *S*. Enteritidis (19.1%) was fivefold that of *S*. Typhimurium (3.7%). In the Democratic Republic of Congo (DRC), 14.9% of iNTS isolates were found to be resistant to azithromycin, all of which were *S*. Typhimurium (22). In Asia, azithromycin resistance rates of 16.1% in India and 18.0% in Vietnam (16) have been reported previously. In Taiwan, 3.1% of NTS isolates were observed to be resistant to azithromycin, with 3.3% of *S*. Typhimurium isolates being resistant to azithromycin compared to 0.4% of *S*. Enteritidis (39). Given that widespread use of azithromycin selects for resistance (39, 40), the increasing resistance of NTS to azithromycin observed in this study could be attributed to overuse of the antibiotic in the health facilities for empiric therapy. The emergence and plausible spread of azithromycin resistance in these settings is a concern as it is likely to limit its use in the management of iNTS and typhoid fever, especially in disease-endemic, resource-limited settings where alternative antibiotics are costly or unavailable.

In this study, a small proportion (8.1%) of the NTS were MDR, which indicates a decline compared to previous findings where resistance to ≥2 antimicrobials in iNTS was >57% in 2019 (4) and 59.3% in NTS in 2020 (13). Our finding also differs from other studies which have reported high MDR rates, though in iNTS, of 81.5% in Malawi (20) and 87.5% in DRC (22). However, recent findings from Malawi indicate a substantial decline in MDR iNTS isolates between 2011 and 2019 for *S*. Typhimurium (78.5%–27.7%) and for *S*. Enteritidis (31.8%–0%) (37). In this study, NTS showed 9.5% and 4.1% resistance to ampicillin and sulfamethoxazole/trimethoprim, respectively. In contrast, a recent study in Nigeria (21) reported that all *S*. Enteritidis and *S*. Typhimurium isolates were resistant to ampicillin and sulfamethoxazole/trimethoprim. Noteworthy in this study is the resistance to third-generation cephalosporins (cefotaxime, ceftriaxone, and cefpodoxime) observed in *S*. Typhimurium isolates. Overall, 4.1% (3/74) of all NTS isolates (all from the same patient) were resistant to the three cephalosporins. A previous study in the same setting reported that 9.1% of NTS were resistant to (cefotaxime or ceftriaxone or ceftazidime) in 2020 (13). The decline in resistance could be attributed to epidemiological changes of NTS strains in this setting, given that all *S*. Typhimurium isolates in this study were of ST19 (29), while in previous studies, a huge proportion were ST313 (13). *S*. Typhimurium ST313 has been mainly MDR and ESBL compared to ST19 (13, 41). In addition, the decline could be attributed to a reduction in the use of these antibiotics and an increase in the use of other antibiotics within this setting. Other studies in Kenya (26, 42) have also reported varying NTS (invasive and gastroenteritis) resistance to ceftriaxone ranging from 2% to 100%. The high susceptibility of NTS to chloramphenicol observed in the present study could be due to the limited use of this antibiotic within this setting.

Although the proportion of MDR isolates was low, 14 different antibiotic resistance determinants were identified in various isolates (Table 1). Carriage of ESBL genes (*bla*_CTX-M-3_, *bla*_TEM-135_, *bla*_TEM-1B_, and *bla*_TEM-1C_) in *S*. Typhimurium isolates could be associated with the resistance to β-lactams observed in the present study. These findings are consistent with observations of previous studies in Kenya, which have reported the detection of *bla*_CTX-M-15_ and *bla*_TEM-1_ genes in β-lactam-resistant NTS (26). However, the *bla_OXA_* gene, which has been reported previously in the current study setting (13), and *bla*_SHV_ were not detected in the current study. Interestingly, while 13.5% (10/74) of the isolates were resistant to azithromycin, the *mph*(A) gene associated with macrolide resistance was present in only one isolate. We hypothesize that enhanced expression of efflux pumps mediating macrolide resistance could be contributing to the reduced susceptibility to azithromycin. Efflux pumps have been shown to be critical in azithromycin resistance (39, 43). In addition, of the 18 isolates exhibiting resistance to tetracycline, only 9 carried the *tetA* gene, which indicates that tetracycline resistance in the other isolates could be due to other mechanisms. The discordance in AMR genotype and phenotype is highlighted in Table S1. We observed NTS carriage of conjugative plasmids such as IncI1_1_Alpha, which harbored multiple resistance genes, including *sul1*, *dfrA1*, *tetA*, and *bla_TEM-1C_*, highlighting the plausible dissemination of resistance genes between species. The detection of epidemic resistance plasmid replicons IncF and IncI demonstrates the plausible spread of resistance, further contributing to the prevalence of MDR strains. This scenario is worsened by the asymptomatic shedding of NTS carrying conjugative plasmids harboring resistance genes, as it could lead to the spread of MDR NTS, particularly in this study setting where poor sanitation and hygiene, open sewers with fecal waste, and improper disposal of solid waste are common.

In this analysis, we identified several virulence genes underscoring the pathogenicity of the NTS isolates. None of the isolates in this study harbored SPI-4 genes encoding type 1 secretion system, a finding that was also observed recently in a study in Nigeria (21). The absence of these genes does not seem to affect the virulence of either *S*. Typhimurium or *S*. Enteritidis. Importantly, one *S*. Typhimurium isolate, which was missing five virulence genes (*avrA*, *rcK*, *spvB*, *spvC*, and *spvR*), present in all the other isolates, carried the nonfimbrial adherence determinant (*shdA*), which has been shown to be critical in fecal shedding (44). However, the isolate was recovered during acute disease and not during shedding. The detection of the *gogB* effector gene demonstrates the role of phage lysogenic conversion in the dissemination of virulence genes into *Salmonella*. GogB has been identified as an anti-inflammatory effector that limits host tissue damage, enabling bacteria to reach optimal densities during infection (45). Given the variance in virulence determinants in some isolates, there is a need for further molecular investigation of virulence factors to understand NTS pathogenicity.

In conclusion, an increase in resistance to azithromycin, third-generation cephalosporins, and ciprofloxacin is likely to limit the treatment options for iNTS and typhoid fever, which could contribute to treatment failure and poor patient outcomes. Consequently, it is imperative to develop integrated prevention and control measures (improved sanitation and hygiene infrastructure), including vaccine implementation. In addition, these findings underscore the need for laboratory surveillance and importance of antimicrobial stewardship to help inform empiric therapy in primary healthcare facilities where there are limited laboratory testing services. Currently, sampling and data generation are limited to a few studies that show large differences in antibiotic resistance patterns. The setup of a continuous integrated genomic surveillance of NTS circulating in endemic settings is critical, considering that the epidemiology of circulating strains could change within a short period. There is also a need for more studies on SPIs of NTS to further elucidate pathogenesis, as this could be crucial in advancing prevention and treatment measures.

## Data Availability

Sequence data of assemblies is available through BioProject ID PRJNA1153302 in the National Center for Biotechnology Information Data Libraries (GenBank).

## SUPPLEMENTAL MATERIAL

Table S1 spectrum.00985-25-s0001.xlsx Antimicrobial phenotypes and genotypes of nontyphoidal *Salmonella*.

ASM does not own the copyrights to Supplemental Material that may be linked to, or accessed through, an article. The authors have granted ASM a non-exclusive, world-wide license to publish the Supplemental Material files. Please contact the corresponding author directly for reuse.
